# Serotype distribution and antibiotics susceptibility pattern of *Streptococcus pneumonia* in Iran

**DOI:** 10.5812/ircmj.8053

**Published:** 2013-10-05

**Authors:** Samira Habibian, Ali Mehrabi-Tavana, Zyanab Ahmadi, Morteza Izadi, Nematolah Jonaidi, Jalalodin Darakhshanpoure, Mahmode Salesi, Seyed Mohsen Zahraei, Ramezan Ali Ataee

**Affiliations:** 1 Department of Medical Microbiology, Tonokabon Azad University of Medical Sciences, Tonokabon, IR Iran; 2 Health Management Research Center, Department of Medical Microbiology, Faculty of Medicine, Baqiyatallah University of Medical Sciences, Tehran, IR Iran; 3 Molecular Biology Research Center, Baqiyatallah University of Medical Sciences, Tehran, IR Iran; 4 Health Research Center, Baqiyatallah University of Medical Sciences, Tehran, IR Iran; 5 Ministry of Health and Medical Education, Center for Communicable Disease Control, Tehran, IR Iran; 6 Department of Medical Microbiology, Faculty of Medicine, Baqiyatallah University of Medical Sciences, Tehran, IR Iran

**Keywords:** Antimicrobial susceptibility, Streptococcus pneumonia, Iran

## Abstract

**Background:**

The development of antibiotic resistance among *Streptococcus pneumoniae* strains has caused significant health problems worldwide.

**Objectives:**

The aim of this study was to determine antibiotic resistance pattern and serotypes distribution of *Streptococcus pneumoniae* strains isolated from clinical specimens.

**Material and Methods:**

A total of fifty *Streptococcus pneumoniae* strains were isolated from Tehran Hospital’s laboratory from 2008 to 2012. Antimicrobial susceptibility testing was performed using broth microdilution method and minimum inhibitory concentration (MIC) of each strain was determined. to verify the resistant strains and demonstrate the presence of antibiotic resistant genes, the PCR was performed.

**Results:**

The study showed that three strains (6%) and six strains (12%) indicated intermediate resistance and complete resistance to penicillin, respectively, 58% strains were susceptible to ceftazidime, two ones (4%) indicated resistance to ciprofloxacin, one (2%) indicated intermediate resistance to ceftriaxone , two strains (4%) indicated complete resistance and four (8%) strains indicated resistance to vancomycin.

**Conclusions:**

The emergence of *Streptococcus pneumoniae* strains with multiple resistance needs permanent monitoring of antibiotic susceptibility patterns of clinical isolates. We have found that ceftazidime is not a suitable drug for choosing the treatment of pneumococcal infections.

## 1. Background

*Streptococcus pneumoniae* (*S. pneumoniae*) is a major invasive pathogen that is the most common cause of community-acquired pneumonia (CAP) (1). Despite the implementation of vaccination programs in children and the elderly, Pneumococcal infections still remain as a major cause of death, especially in children worldwide (2, 3). Based on the reports, annually, pneumococcal infections leave one to two million people dead worldwide (4, 5). This pathogen causes deadly infections such as meningitis, pneumonia, otitis media, bacteremia both in developing and developed countries (6). Based on capsular polysaccharide composition, *S. pneumoniae* strains are classified into forty serogroups and more than ninety serotypes. However, the prevalence of serotypes varies in different geographical areas, whereas pneumococcal infections are caused by only a limited number of serotypes (4, 7). In addition, the development of antibiotic resistance among these bacterial strains causes important health problems worldwide (8). In these cases, the development of antibiotic resistance in *S. pneumoniae*, causes complexity of treatment and management of Pneumococcal infections. Therefore, not only the delays in treatment, but also the increased health costs have also been created (9, 10). Recent studies have shown that *S. pneumoniae* is one of the five pathogenic Gram-positive bacteria that demonstrate the significant levels of antibiotic resistance (8). However, still in some countries, penicillin is the first choice for treatment of pneumococcal infections, resistance to Penicillin was rigorously reported in the last decade (11, 12). In recent years, the spread of *S. pneumoniae* strains that is resistant to more than one antibiotic has increased in many countries (13). In one study in Canada, resistance to penicillin in *S. pneumoniae* strains increased up to 1.7%, on average (14). Another study in Belgium reported penicillin-resistant pneumococccus increased from 2.3 % in 1993 to 7.6% in 1994 (15). Moreover, a study in Brazil revealed Pneumococcal resistance to Penicillin increased from 9.6% in 1993 to 20.6% in 1996 (16). In addition, the emergence of resistance in other antibiotics classes has increased rapidly (4). Recent report declared that 15 to 30% (OF) isolated strains of *S. pneumonia *have shown multidrug-resistant (MDR) (i.e., resistant to≥ 3 classes of antibiotics) (17, 18).

## 2. Objectives

As a consequence, the emergence of *S. pneumoniae* strains with multiple-resistance needs permanent monitoring of antibiotic susceptibility patterns of clinical specimens. The aim of this study was to determine the antibiotic resistance pattern and measurement of minimum inhibitory concentration (MIC), of the serotypes determined, *S. pneumoniae* strains isolated from clinical specimens.

## 3. Materials and Methods

### 3.1. Materials

#### 3.1.1. Antimicrobial Agent and Culture Media

Antimicrobial agents used in this study consist of penicillin G (800,000 I.U/Vial) (Jaber Ebne Hayyan Pharmacological Co, Iran), Ciprofloxacin (200 mg/100mL) (KRKA, Slovenia), ceftazidime (1g/ Vial) (EXIR Pharmacological Co Iran), ceftriaxone (1g/ Vial) (Jaber Ebne Hayyan Pharmacological Co, Iran), vacomycin (500mg/ Vial) (Dana Pharmacological Co, Iran). Mueller-Hinton broth (Merck, Germany), defibrinated sheep blood (Baharafshan, Iran), 96-well microplate (from Technogen) and injectable distilled water.

#### 3.1.2. Bacterial Strains and Serotype Determination

A total of fifty *S. pneumoniae* strains were isolated from Tehran Hospital’s laboratory from 2008 to 2012. In a previous study, all isolated strains were identified and their serotypes were determined and classified. They were then lyophilized and stored at -80^o^C.

### 3.2. Methods

In this study, based on CLSI and EUCAST criteria ( 19 , 20 ) and the set up method, from each selected antibiotic, serial dilutions were made from stocks solutions and then they were loaded in 96-well plates ( 21 ). The inclusion criteria were based on CLSI and EUCAST, which refer to the minimum inhibitory concentration of selected antibiotics (Table 1), isolated strains were classified into susceptible, intermediate and resistant.

**Table 1.** CLSI and EUCAST criteria for Minimum Inhibitory Concentration MIC (µL/mL) of Streptococcus pneumonia

Antimicrobial Agent	MIC (µg/mL) Interpretive Standard		
	Susceptible	Intermediate	Resistant
**Penicillin**			
**Nonmeningitis**	2	4	8
**Meningitis**	0.5	1	2
**Ciprofloxacin**	1	2	4
**Ceftazidime**			
**Nonmeningitis**	1	2	4
**Meningitis**	0.5	1	2
**Ceftriaxone**			
**Nonmeningitis**	1	2	4
**Meningitis**	0.5	1	2
**Vacomycin**	1	-	-

#### 3.2.1. Microplate designing

Designing of the 96-well microplate was performed as follows, for preparation of the serial dilutions of selected antibiotics, a stock solution of each antibiotic was prepared. In a 96-well microplate; one row for negative control, one row for positive control and three rows for tests were marked, respectively. Thus, each 96-well microplate was considered to measure the antibiotic sensitivity of two bacterial strains. The negative control rows for each case consist of the serial dilutions of the antibiotic, only culture media (Mueller-Hinton broth containing 3% sheep blood) and lacking bacterial suspension. The positive control rows consist of culture media (Mueller-Hinton broth containing 3% sheep blood) and the bacterial suspension. The positive control rows lack an antibiotic. The three rows of tests with equal conditions were used for each bacterial strain testing. The test rows composed of serial dilutions of antibiotics, culture media and bacterial suspension.

#### 3.2.2. Serial Dilution Preparation of Antibiotics

The stock solutions of each antibiotic were prepared. Different concentrations of antibiotic were then prepared and loaded into defined rows of 96 well microplates from the base antibiotic stock solution. Plates were incubated at 35°C for 24 hours until evaporation and finalization of the process of loading the antibiotic. The plates were stored in a refrigerator set at 4°C whenever required. In this study, ten serial dilutions were defined for penicillin. The stock was prepared from penicillin G (800,000 I.U/ vial) so that each 1µL volume of stock contained 40 µg of antibiotic. Seven serial dilutions were defined for ciprofloxacin. The stock was prepared from ciprofloxacin (200 mg/100mL/ vial) so that each 1µL volume of stock contained 2µg of antibiotic. Ten serial dilutions were defined for ceftazidime. The stock was prepared from ceftazidime (1g/ vial) so that each 1µL volume of stock contained 40 µg of antibiotic. The stock was prepared from ceftriaxone (1g/vial) so that each 1µL volume of stock contained 10µg of antibiotic. Eight serial dilutions were defined for ceftriaxone. Finally nine serial dilutions were defined for vancomycine. The stock was prepared from the vancomycin vial (500mg) that each 1µL volume of stock contained 20 µg of antibiotic. The details of the serial dilutions prepared for each antibiotic are presented in Table 2.

**Table 2.** An example of a row of a 96-Wells microplate used for the antibiotic susceptibility pattern using the broth microdilution method for each antibiotic

		1	2	3	4	5	6	7	8	9	10	11	12
**penicillin**	A	O	O	O	O	O	O	O	O	O	O	O	O
	µg	25	50	75	100	125	150	175	200	250	300		
	µL	0.625	1.25	1.875	2.5	3.125	3.75	4.375	5	6.25	7.5		
**ciprofloxacin**	A	O	O	O	O	O	O	O	O	O	O	O	O
	µg	1.5	3.125	6.15	12.5	25	50	100					
	µL	0.75	1.5	3.125	6.25	12.5	25	50					
**ceftriaxone**	A	O	O	O	O	O	O	O	O	O	O	O	O
	µg	0.175	0.35	7.5	15	30	60	120	240				
	µL	0.175	0.35	0.75	1.5	3	6	12	24				
**ceftazidime**	A	O	O	O	O	O	O	O	O	O	O	O	O
	µg	25	50	75	100	125	150	175	200	225	250		
	µL	0.625	1.25	1.875	2.5	3.125	3.75	4.375	5	5.625	6.25		
**vacomycin**	A	O	O	O	O	O	O	O	O	O	O	O	O
	µg	2	4	7.75	15.5	31.25	62.5	125	250	500			
	µL	0.1	0.2	0.387	0.775	1.562	3.125	6.25	12.5	25			

#### 3.2.3. Preparation of Bacterial Inoculums

Based on CLSI standard, using the broth microdilution method, antimicrobial susceptibility testing was carried out. For this purpose, the turbidity of bacterial suspension equivalent to McFarland 0.5 turbidity standard which represents 1.5×108 bacteria/mL was prepared. A colony of each bacterium was inoculated into a BHI broth medium containing 3% sheep blood separately, and incubated at 37^o^C. When the absorption rate of bacterial suspension was equivalent to McFarland 0.5 turbidity standard in 620 Nanometer wavelengths, it was used as the bacterial inoculums.

#### 3.2.4. Antimicrobial Susceptibility Test

As aforementioned, each antibiotic was loaded in triplicates row and employed for each serial dilution testing in the 96-well microplate. In the same manner, one row of the 96-well microplate was considered as a negative control for each antibiotic and one row of the 96-well microplate was considered as a positive control which consists of culture medium and bacterial suspension. A volume of 150µL Mueller-Hinton broth containing 3% sheep blood was added to each well and was shacked for 30 seconds. Finally, 50µL of bacterial inoculums with McFarland 0.5 turbidity standard were added to wells.

In this study, a daily maximum of four bacterial strains were tested. For each case, the pre- loaded plates were removed from the refrigerator and 150 μL of Mueller-Hinton broth containing 3% sheep blood was added to each well. The negative control well or blank did not contain bacterial inoculums, whereas the positive control wells were free from antibiotics. The McFarland 0.5 standard provides turbidity (OD = 7) comparable to that of a bacterial suspension containing 1.5 ×108 colony-forming units (CFU)/mL or 1.5 × 105 CFU/μL. The turbidity of the prepared bacterial suspensions was compared by observing the black lines through the suspension. A 50μL volume of this suspension was added to each well to obtain a final concentration of inoculums, which was approximately 50 × 1.5 × 105 CFU/wells. Two bacterial strains were tested in a plate separately. Inoculated plates were incubated at 37°C, and the optical density (OD) of each well was measured at time 0 and 24 hours after initiation of the incubation using an ELISA reader device set at 450 Nanometer. The mean of the OD of different concentrations of antibiotics per well for each bacterium was compared and analyzed using unilateral variance analysis (ANOVA). In our statistical analyses, α = 0.005 was considered acceptable significant variation and the results were analyzed using SPSS Ver. 16.

#### 3.2.5. Molecular Assay for Pbp2b

Polymerase chain reaction amplified the fragment pbp2b gene. This was carried out by using the primer pair’s sequences: F- 5' GAT CCT CTA AAT GAT TCT CAG GTG G 3' and R- 5' TGG TGT TCG TGT GGC TCC TC 3'. The Pbp2b gene is a major gene, causing resistance to Penicillin in *S. pneumoniae*. In our study, the mentioned primers pair were selected and synthesized by CinnaGen Co Iran. In purpose of detection and amplification of pbp2b gene, overnight growth bacterial cells of an exponential phase were subjected to DNA extraction using the salting out method (22). We prepared PCR reaction master mixing in 25µL volume in 0.2mL micro centrifuge tubes. An individual reaction was contained in the following: 2.5µL PCR buffer (10x), 0.5µL MgCl2 (50mM), 0.5µL dNTPs (10mM), 0.5 µL of each primer (100pm/µL), 0.5 µL Taq DNA polymerase (5u/µL), 1.5 µL template DNA and 18.5 µL sterile and deionized H2O. All the materials used in PCR reactions were purchased from CinnaGen Co Iran. Thermal cycling was performed in the Antaltik Jena PCR system. The PCR process included: 94°C for 6 min followed by 35 amplification cycles of 94°C for 45s, 61°C for 30s, 72°C for 1 min and with 5 min final extinction at 72°C. For the analysis of PCR products, a 1.5% agarose gel containing 1X TBE buffer was prepared and run at 100 V for 40 min. For determining the size of PCR products, we used a 1kb DNA ladder (Fermentase). The size of the PCR product of pbp2b gen was about 1500bp.

## 4. Results

The results of this study suggest that the antibiotics’ concentrations loaded in the 96-well microplates could keep their activity at least for six months, because during this time no changes were observed in antimicrobial activity of the antibiotics. An example of data analyzing the results of antimicrobial susceptibility testing is shown in Table 1 and its significance in Table 2. In each 96 well microplate, one row was considered as a negative control (containing serial dilutions of antibiotics and culture media). One row was considered as a positive control (containing culture media and a similar volume of bacterial inoculums) and three rows that were the same considered as tests for susceptibility testing of bacterial strains. After inoculation and incubation of the microplates, they were scanned with ELISA reader device (TECAN) set at 450 Nanometer wavelengths and the absorbance of each well was documented. For each strain, negative control wells were defined with code 1, positive control wells were defined with code 2 and three rows of test wells were defined as code 3 and were analyzed using SPSS Ver. 16.0 In all cases, the average of the triple wells’ optical densities (ODs) of one antibiotic’s concentration was subtracted from the negative control’s ODs. Finally, the results were compared with the positive control’s ODs. The output of software was about fifty pages of A4 paper sheets. In continue, the data analyzing results of one susceptible strain (Table 3) and one resistant strain (Table 4) of *S. pneumoniae *to penicillin have been mentioned. 

**Table 3.** Data analysis of the susceptibility testing of a sensitive (Code 208) and resistant (Code 108) strains of*Streptococcus pneumoniae*in different penicillin concentrations

A)*Streptococcus pneumoniae*serotype 6 as a sensitive Penicillin strain (Code 208)								
Penicillin Concentration	Num	Mean OD	Std. Deviation	Std. Error	%95 Confidence Interval for Mean OD		Minimum	Maximum
					Lower Bound	Upper Bound		
**0^a^**	10	1.05820	0.075332	0.023822	1.00431	1.11209	1.016	1.270
**25**	3	0.71450	0.084184	0.048604	0.50537	0.92363	0.617	0.769
**50**	3	0.63233	0.097449	0.056262	0.39026	0.87441	0.528	0.721
**75**	3	0.69367	0.085979	0.04940	0.48008	0.90725	0.600	0.769
**100**	3	0.61733	.0.041016	.0.023681	.0.51544	0.71922	0.587	0.664
**125**	3	0.69817	0.049410	0.028527	0.57543	0.82091	0.646	0.744
**150**	3	0.85733	0.010017	0.005783	0.83245	0.88222	0.846	0.865
**175**	3	0.66550	0.028827	0.16643	0.59389	0.73711	0.635	0.691
**200**	3	0.64217	0.042253	0.024395	0.53720	0.74713	0.598	0.682
**225**	3	0.65183	0.032655	0.018853	0.57071	0.73295	0.632	0.690
**250**	3	0.63383	0.037448	0.021620	0.54081	0.72686	0.594	0.668
**Total**	40	0.77505	0.183860	0.029071	0.71625	0.83385	0.528	1.270
**B) serotype 14 as a Resistant Penicillin strain (Code 108)** ***Streptococcuspneumoniae*****serotype 14 as a Resistant Penicillin strain (Code 108)**								
**0 ** ^**a**^	10	0.89730	0.297466	0.094067	0.68451	1.11	1.1	1.247
**25**	3	0.88750	0.101868	0.058813	0.63445	1.140	0.771	0.956
**50**	3	0.51167	0.007095	0.004096	0.49404	0.52929	0.504	0.518
**75**	3	0.51017	0.028989	0.016737	0.43816	0.58218	0.487	0.543
**100**	3	0.50600	0.020298	0.011719	0.45555	0.55642	0.488	0.28
**125**	3	0.50433	0.058227	0.033617	0.35969	0.64898	0.438	0.547
**150**	3	0.47417	0.036295	0.020955	0.384	0.56433	0.436	0.508
**175**	3	0.47183	0.034152	0.019717	0.387	0.55667	0.435	0.502
**200**	3	0.45550	0.127330	0.073514	0.35701	0.57181	0.409	0.503
**225**	3	0.44167	0.034078	0.019675	0.29823	0.52632	0.396	0.477
**250**	3	0.42883	0.052577	0.03035	0.13919	0.55944	0.380	0.400
**Total**	40	0.61370	0.249111	0.039388	0.53403	0.69337	1.1	1.247

^a^ 0 in equal positive control which lake of serial concentration of antibiotic

**Table 4.** Significance of data analysis of the susceptibility testing of two*Streptococcus pneumoniae*strains ^a ^

A)*Streptococcus pneumoniae*serotype 6 as a sensitive Penicillin strain (Code 208)	Penicillin Con			Mean Difference	Std. Error	Sig.
**%59 Confidence Interval**		**Test Wells (T)**	Positive Control Well (PC)		(PC) – (T)	
	Lower Bound	Upper Bound	**25**	0		−0.3437
0.41927	0.000	−048898	−0.21842	**50**	0	
−0.425867	0.41927	0.000	−0.55114	−0.30059	**75**	0
−0.364533	0.41927	0.000	−0.48981	−0.23926	**100**	0
−0.440867	0.41927	0.000	−0.56614	−0.31559	**125**	0
−0.360033	0.41927	0.000	−0.48531	−0.23476	**150**	0
−0.200867	0.41927	0.000	−0.32614	−0.07559	**175**	0
−0.392700	0.41927	0.000	−0.51798	−0.26742	**200**	0
−0.416033	0.41927	0.000	−0.54131	−0.29076	**225**	0
−0.406367	0.41927	0.000	−0.53164	−0.28109	**250**	0
−0.424367	0.41927	0.000	−0.54964	−0.29909	**P≤ 0.000**	**B)Streptococcuspneumoniaeserotype 14 as a Resistant Penicillin strain (Code 108)**
**25**	0	−0.0098	0.114133	1.000	−0.35083	
0.33123	**50**	0	−0.385633	0.114133	0. 19	
−0.73399	−0.04461	**75**	0	−0.387133	0.114133	
0. 19	−0.72816	−0.4611	**100**	0	−0.391300	
0.114133	0. 17	−0.72333	−0.05027	**125**	0	
−0.392967	0.114133	0. 16	−0.73399	−0.05194	**150**	
0	−0.425467	0.114133	0.08	−0.76649	−0.08444	175
0	−0.423133	0.114133	0.08	−0.76416	−0.08211	200
0	−0.4418	0.114133	0.05	−0.78283	−0.10077	225
0	−0.455633	0.114133	0.04	−0.79666	−0.11461	250
0	−0.468467	0.114133	0.03	−0.80949	−0.12744	

^a^P ≤ 0.05 is significant

The results of the serotypes distribution and antimicrobial susceptibility of 50 strains of *S. pneumoniae *are shown in Table 5. Based on our findings, penicillin MICs less than 75U/mL were considered as susceptible strains. MICs more than 75U/mL to 100U/mL were considered as intermediate strains and MICs higher than 100U/mL were considered as resistant strains. As a result, only three strains (6%) have shown intermediate resistance to penicillin. One strain belonged to serotype 1 and others belonged to serotype 4 that were isolated from a lung infection. The other intermediate strain belonged to serotype 20 was isolated from CSF. In addition, six strains (12%) have shown complete resistance with MIC higher than 125U/mL. These strains belonged to serotypes 7, 14 and 20 and all of them were isolated from the throat. 

**Table 5.** MIC Penicillin and Serotype Distribution

Origin	Internal Code Number	Serotype or Serogrope	Minimal inhibitory Concentration Penicillin									
			MIC ≤ 25U/mL	MIC ≤ 50U/mL	MIC ≤ 75U/mL	MIC ≤ 100U/mL	MIC ≤ 125U/mL	MIC ≤ 150U/mL	MIC ≤ 175U/mL	MIC ≤ 200U/mL	MIC ≤ 225U/mL	MIC ≤ 250U/mL
**Wounds**	104, 105	6, 5		50U/mL								
	381	10			75U/mL							
**Rhinit**	778	7; 7		50U/mL								
	49		25U/mL									
**Sinus**	115	20		50U/mL								
**Eye**	101; 208	6;6	25U/mL									
	7; 996; 14; 5; 502	19; 6; 14; 6; 19		50U/mL								
	129; 50; 53	6; 19; 19			75U/mL							
**Throat**	128; 123	7; 20										250U/mL
	103; 108	14;14										250U/mL
**Lung**	777	1	25U/mL									
	125; 501	1; 4				100U/mL						
	6; 117; 202; 107; 106; 130; 83; 102; 120; 201; 48; 13; 28	7; 7; G;										
	124	8; 1; 2; 2; 6; 6; 6; 8; 6; 8					125U/mL					
	34	2		50U/mL								
**blood**	116; 121; 35; 40	8										
	126; 133	4; 6; 18; 17	25U/mL									
	131; 89	17; 1		50U/mL	75U/mL							
	39	6; G			75U/mL							
**CSF**	109	2			75U/mL							
	111	20				100U/mL						
	51	20					125U/mL					

**Figure 1.**
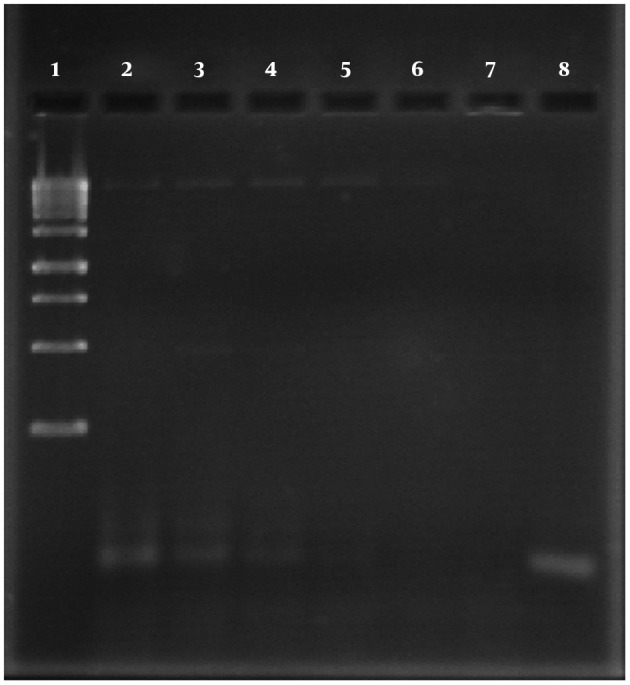
the results of PCR product electrophoresis for pbp2b gene. Line 1 shows the Ladder 1kb. Lines 1 - 7 show temperature gradient and PCR product 1500bp. Line 8 is negative control.

Two strains had Penicillin MICs equivalent to 125U/mL and belonged to serotype 8 that isolated from lung and CSF, respectively. Besides, four strains had Penicillin MICs equivalent 250U/ml. These strains were completely resistant to Penicillin. We used the same method for other antibiotics consist of Ciprofloxacin, ceftazidime, ceftriaxone and vacomycin. The only differences were the concentrations used for each antibiotic. In addition, the result of susceptibility testing of *S. pneumoniae *to vancomycin have shown that serotypes 14 (isolated from CSF), and 20 (isolated from throat) had fully resistance. Furthermore, serotypes 17 (isolated from blood) and 20 (isolated from throat) have MICs more than 10µg/mL. The results of molecular detection gene pbp2b which amplified fragment 1500 bp showed that 4 strains revealed the fragment 1500 bp (Figure 1). 

## 5. Discussion

Despite the widespread use of antibiotics, especially Penicillin and discovery of new antimicrobial agents, *S. pneumoniae* remains as one of the deadliest pathogens, especially in children and the elderly. Traditionally, Penicillin is used as the drug of choice in the treatment of Pneumococcal infections. But in recent decades, especially from the 1990s, a reduction in Pneumococcal susceptibility to Penicillin and other antibiotics is observed (23-25). Emergence of *S. pneumoniae* strains with multiple resistance needs permanent monitoring of antibiotic susceptibility patterns of clinical samples (18). The results of this study suggest that the four serotypes were completely resistant to Penicillin: Because the minimum inhibitory concentration was about 250 U/mL of Penicillin. In addition, the results indicated that serotype 20 had a multidrug resistance. This serotype, which had been isolated from a patient’s throat, was resistant to penicillin, ceftazidime and vancomycin.

However, annually, many reports have revealed the development of antimicrobial resistance in *S. pneumoniae*. A study in Spain revealed that resistance to Penicillin , amoxicillin, cefotaxime, tetracycline, chloramphenicol, erythromycin and levofloxacin were 47.5%, 7.8%, 21.3%, 38.3%, 22.1%, 34.8% and 1.5% , respectively (26).

A report in France has shown that susceptibility to penicillin, erythromycin and tetracycline decreased (21%, 57% and 43% of the isolates, respectively) in clinical samples (27). In another study in the United States, it was shown that 30% of *S. pneumonia* strains was multidrug-resistant and also, 30 - 60% of clinical samples were resistant to penicillin (and amoxicillin) (28). Meanwhile, in a study in Nepal from 1999 to 2008, for cotrimoxazole, penicillin, ampicillin, Erythromycin and chloramphenicol, the overall resistance percentage was 56.6%, 4.7%, 5%, 5.3% and 2.4%, respectively with variation in some year (29). In China, a study reported the resistance rates of the *S. pneumoniae* isolates to erythromycin, clindamycin, tetracycline and trimethoprim-sulfamethoxazole were 84.6%, 84.6%, 82.1% and 74.4%, respectively and only 10.3% were susceptible to all tested antimicrobials (30). In a study in Southeast Austria that was performed in 1997 to 2008, it was reported that resistance to erythromycin increased from 3.5% in 1997 to 14.7% in 2008. For trimethoprim/sulfamethoxazole, resistance increased slightly to 9.2% in 2008 (31). Moreover, in Nigeria a study reported an antimicrobial resistant profile of *S. pneumoniae* isolated from suspected tuberculosis patients. Resistance ranges to some selected antibiotic agents were shown in this study as the following: norfloxacin 85.7%, ampicillin 85.7%, erythromycin 71.4%, oxacillin 42.9%, chloramphenicol 28.6%, gentamicin 28.6%, ceftaxidine 14.3%, peflacine 14.3% (32). Results from many studies have shown that despite the control and reduction in the consumption of antibiotics, the resistance to many antibiotic agents is still increasing (33). So, for selection of an appropriate antibiotic agent with regard to the reduction in treatment cost and period of recovery, it seems essential to monitor susceptibility of *S. pneumoniae* for selective antibiotic agents. As a result, choosing a proper and reliable antibiogram method seems essential for analysis of results. In our study, we defined the resistance pattern of *S. pneumoniae*, using broth microdilution method. Broth microdilution MIC test method is a standard method for most bacteria (34). This method is a quantitative reference method which can be used in antibiotic susceptibility testing in comparison with agar-based method; broth microdilution method can decrease much labor and time. In comparison with disk diffusion method, broth microdiution method has this advantage that could determine the MIC value for antibiotic agents. In addition, broth microdiution method is a more reliable and accurate method than disk diffusion method (35). Finally, for verification of the pbp2b gene existence that causes resistance to penicillin, we performed a PCR reaction. This molecular method confirmed the existence of the pbp2b gene in resistant strains that were identified by broth microdilution method. The results of our study are as follows: three strains (6%) showed intermediate resistance to penicillin and six strains (12%) showed complete resistance, 58% of strains were susceptible to ceftazidime, two strains (4%) showed resistance to ciprofloxacin and one strain (2%) showed intermediate , two strains (4%) have shown complete resistance to ceftriaxone, four (8%) strains showed resistance to vancomycin. In conclusion, ceftazidime is not a proper drug for choosing the treatment of Pneumococcal infections.
